# Modulating Casein-Stabilized Emulsions Through the Incorporation of Different Emulsifiers: Impacts on Microstructure and Oral Tribology

**DOI:** 10.3390/foods15050846

**Published:** 2026-03-03

**Authors:** Shujun Ji, Ping Liu, Mengya Sun, Mengmeng Xu, Xiaojie Zhang, Qiongyu Wang, Zhihua Pang, Xinqi Liu

**Affiliations:** Key Laboratory of Geriatric Nutrition and Health, National Soybean Processing Industry Technology Innovation Center, School of Food and Health, Beijing Technology and Business University (BTBU), Beijing 100048, China; jishujun77@163.com (S.J.); liuping970928@163.com (P.L.); sunmengya66@163.com (M.S.); 13613971681@163.com (M.X.); m18706553135@163.com (X.Z.); sykuangyou@163.com (Q.W.); liuxinqi@btbu.edu.cn (X.L.)

**Keywords:** casein, emulsion, emulsifier, saliva, tribology

## Abstract

This study investigated the combined effects of oil concentration, emulsifier type, and saliva on the lubrication behavior of casein-based oil-in-water emulsions to support the design of milk-based foods with optimized mouthfeel. Emulsions stabilized with Tween 20, whey protein isolate (WPI), or sucrose ester were prepared at oil concentrations ranging from 0.01% to 3%, and their viscosity, microstructure, tribological properties, and ζ-potential were systematically characterized, with human saliva incorporated to simulate oral conditions. Oil concentration did not significantly alter viscosity, although droplet aggregation increased with higher oil levels. Lubrication performance was governed primarily by emulsifier type: Tween 20 generated an oil film at approximately 0.2% oil, WPI exhibited progressively enhanced lubricity with increasing oil concentration, and sucrose ester produced consistently poor lubrication due to its rigid interfacial layers. Saliva addition improved lubrication across all systems and reduced oil precipitation by promoting the formation of smaller, more stable structures. These findings demonstrate that emulsifier selection is central to modulating oil–protein–saliva interactions, with WPI at moderate oil levels yielding favorable lubrication with controlled oil release, thereby providing a mechanistic basis for developing healthier, palatable milk-based foods.

## 1. Introduction

Food emulsions, integral to everyday products such as milk, ice cream, and sauces, constitute a fundamental class of colloidal systems whose stability and sensory properties are central to food science and industrial applications [1]. The lubricating properties of food are determined by the complex interactions between its specific composition, microstructure, and physical state. For example, dairy systems with higher fat content typically exhibit superior lubricating characteristics [2]. Beyond the simple volume fraction of oil, the mechanical stability and coalescence behavior of emulsion droplets are also decisive factors; notably, O/W emulsions prone to coalescence have been reported to yield a lower friction coefficient compared to their more stable counterparts [3]. Smooth, creamy, or velvety mouthfeel—highly valued sensory attributes—primarily arise from the frictional interactions between the food and soft oral tissues [4,5]. Since the pioneering work of Kokini et al. [6], tribology has been increasingly employed to decode the texture perception of fluid and semi-solid foods, enabling a more mechanistic understanding of oral lubrication [7,8,9,10].

Upon ingestion, emulsified foods undergo immediate dilution and interaction with saliva, which alters the concentration and compositional gradient of the system [11]. These emulsions simultaneously function as lubricants, reducing frictional forces between oral surfaces [12]. For the food industry, establishing robust correlations among product composition, microstructure, rheological-tribological behavior, and sensory outcome is therefore essential for rational design and optimization [13,14]. Decades of research have been dedicated to linking oral lubricity with food structure, revealing significant correlations between physical properties and perceptual qualities [15,16,17,18]. In this context, tribology has emerged as a vital tool, with the friction coefficient—often represented via Stribeck curves—serving as a key parameter for elucidating oral lubrication mechanisms [15,18].

The oral behavior of emulsions, especially those stabilized by proteins, is largely dictated by dynamic interactions between salivary components (notably mucins) and the adsorbed layer at the droplet interface [19]. Conventional O/W emulsions are stabilized by surface-active molecules such as proteins and/or low-molecular-weight emulsifiers, whose functionality depends on their amphiphilicity and film-forming ability at the oil–water interface [20,21]. These interfacial assemblies can be further modulated through competitive adsorption or the formation of composite structures via electrostatic, hydrophobic, or hydrogen-bonding interactions, ultimately governing the emulsion’s macroscopic stability and breakdown pathway [5].

Saliva is a complex biofluid comprising over 200 proteins in an aqueous medium, which plays indispensable roles in hydration, lubrication, and interfacial modification in the mouth [11,22,23]. Salivary mucins not only contribute to lubrication but also exhibit emulsifying activity, potentially facilitating the formation of in-mouth emulsion structures during mastication [24]. Consequently, the oral performance of protein-stabilized emulsions is profoundly shaped by the interaction between saliva-derived molecules and the interfacial architecture [19].

Despite these insights, the lubrication characteristics of emulsions are not attributable to any single factor but result from a synergistic combination of oil droplet size distribution, interfacial composition, continuous phase rheology, and salivary effects [25,26]. In particular, the role of saliva extends beyond simple dilution; its components can adsorb onto droplet surfaces, modifying interfacial charge and rheology, and may even re-emulsify free fat, thereby altering frictional behavior [27]. However, a systematic and mechanistic understanding of how emulsifier type modulates the interfacial dynamics—and subsequently the lubrication performance—in casein-based emulsions with varying oil levels remains elusive. The competitive adsorption between emulsifiers and casein, followed by the interplay with salivary constituents, forms a complex sequential process that has yet to be fully unraveled.

To address this gap, the present study aims to systematically investigate the synergistic effects of oil concentration (0.01–3%), emulsifier type (Tween 20, whey protein isolate, sucrose ester), and saliva on the lubrication properties of casein-stabilized emulsions. We hypothesize that the choice of emulsifier governs the interfacial structure and its stability, which in turn regulates the kinetics of oil release and the nature of casein–saliva interactions during oral processing, thereby defining the ultimate tribological and sensory response. Utilizing an integrated methodology encompassing rheology, tribology, confocal laser scanning microscopy, and ζ-potential analysis, this work will (i) examine the influence of oil concentration on emulsion viscosity, microstructure, and friction profiles; (ii) elucidate the competitive adsorption relationships between casein and different emulsifiers at the interface; and (iii) delineate the role of saliva in emulsion breakdown, oil release, and potential re-emulsification. The findings are anticipated to provide a foundational framework and practical design strategies for developing novel, reduced-fat dairy-based products that do not compromise on sensory appeal.

## 2. Materials and Methods

### 2.1. Materials

Soybean oil was purchased from Cofco Fu Linmen Food Marketing Co., Ltd. (Shanghai, China).Tween 20, sucrose ester (HLB 10) and sodium hydroxide were purchased from Fuchen Chemical Reagent Co., Ltd. (Tianjin, China). Casein micelles (protein content 85%) were purchased from Senlang Biochemical Co., Ltd. (Shaanxi, China); polydimethylsiloxane (PDMS) was purchased from Dow Corning Company (Midland, MI, USA). The gold chip (QSX 301) was purchased from Biolink Technologies (Sandnes, Sweden). Hydrochloric acid, 1,2-propanediol and ethanol were purchased from National Pharmaceutical Group Chemical Reagents Co., Ltd. (Shanghai, China). Nile Red was purchased from McLean Biotechnology Ltd. (Shanghai, China).

### 2.2. Preparation of Emulsions

An 8% (*w*/*w*) whey protein isolate (WPI) solution was prepared in ultrapure water and was mechanically stirred for 30 min at 25 °C and 500 rpm using a thermostatic magnetic stirring water bath (SHJ, Jin tan Liang you Instrument Co., Ltd., Changzhou, China). A 1% (*w*/*w*) Tween 20 solution was prepared and mechanically stirred for 30 min at 25 °C and 500 rpm using a thermostatic magnetic stirrer (EUROSTAR 40 D S025, IKA GmbH, Staufen, Germany).

The pH of the WPI solution was adjusted to 7.0 with pre-prepared 1.0 M and 0.1 M HCl or NaOH solutions, and the solution was then stored overnight at 4 °C. Subsequently, an 8% (*w*/*w*) sucrose ester solution was prepared in ultrapure water and was stirred under the same mechanical conditions (500 rpm, 30 min) at 60 °C in a thermostatic magnetic stirring water bath.

Three types of emulsifiers—WPI solution, sucrose ester solution, and Tween 20—were prepared and were separately added at 2% (*w*/*w*) into a mixed system containing 40% soybean oil. The coarse emulsions were first processed using a high-speed dispersing homogenizer (FJ200-SH, Huxi Industrial Co., Ltd., Shanghai, China) at 10,000 rpm for 3 min, and then further homogenized with a high-pressure homogenizer (D-3L, PhD-Tech, Inc., Saint Paul, MN, USA) at 5000 psi. Finally, three distinct oil-in-water (O/W) emulsions were obtained.

### 2.3. Preparation of the Casein System

A 4% *w*/*v* casein micelle solution was prepared by dissolution in ultrapure water. The solution was stirred in a magnetic stirrer water bath at 25 °C and 500 r/min for 4 h, followed by sonication (KQ-500DE, Ultrasonic Instrument Co., Ltd., Kunshan, China) at 300 W for 20 min. It was then stored overnight in a 4 °C incubator for subsequent use. The pre-prepared casein aqueous solution was retrieved and diluted to a 3% concentration. The oil was added to the diluted casein solution to make three different O/W emulsions with oil content of 0.01%, 0.05%, 0.2%, 1%, and 3% in the final system. Each sample was stirred at 500 r/min for 3 min using a laboratory stirrer and then homogenized once at 5000 psi with a high-pressure homogenizer to obtain the final casein emulsion. The casein solution without added emulsion was designated as the control group.

### 2.4. Viscosity Analysis

The viscosity of the samples was determined using a DHR-1 rheometer (TA Instruments, New Castle, DE, USA). Measurements were conducted under the following conditions: a temperature of 37 °C, a stainless steel cone-plate geometry (diameter: 40 mm, cone angle: 2°), a gap of 57 μm, and a shear rate range from 3 to 1000 s^−1^. The variation in viscosity with shear rate was recorded. Approximately 700 μL of sample was dispensed onto the sample stage. The gap was initially set to 59 μm, trimmed, and then adjusted to 57 μm before the measurement was started. Three independent replicates were performed for each sample, and the results are reported as mean values with standard deviations.

### 2.5. Confocal Laser Scanning Microscope

The distribution of soybean oil in different samples was observed using a laser confocal microscope. A 0.2% Nile red solution was prepared by adding Nile red dye to 1,2-propylene glycol. The samples were stained with the Nile red solution at a concentration of 0.05% by mass of Nile red. Samples were prepared in the dark, after which a small amount was applied to a microscope slide and covered with a coverslip. All images were captured using a 40× objective lens, with fluorescence images acquired at an excitation wavelength of 530 nm.

### 2.6. Analysis of Oral Friction Behavior

The lubricating properties of the samples were measured using a Three Ball Rheometer (TA Instruments, USA). Polydimethylsiloxane (PDMS, surface roughness (Ra) < 50 nm; contact angle: 95.33°) was prepared as described in the product manual and employed as the lower contact surface to simulate the tongue. PDMS and crosslinker were mixed at a 10:1 ratio, evacuated to remove entrapped air, and cured at 70 °C for 10 h. Prior to each test, the surfaces were cleaned with ultrapure water. Measurements were conducted at 37 °C under a constant normal force of 1 N. For each test, 4 mL of sample was gently applied to cover the substrate, and the coefficient of friction was recorded over a drag speed range of 0.01 to 30,000 μm/s. Three independent replicates were performed for each sample, and the results are reported as mean values with standard deviations.

### 2.7. Saliva Collection

Saliva was collected according to previously established research protocols [28,29]. Ten volunteers (five males and five females, all aged between 20 and 29 years) were selected, all of whom possessed good dental health and had no history of smoking or other medical conditions. The volunteers were trained in advance and were instructed not to consume anything other than water for one hour prior to saliva collection. Collection was scheduled between 9:30 and 11:30 a.m. Prior to sampling, the volunteers were required to rinse their mouths with pure water for 10 s, and this rinsing procedure was repeated three times. With the tongue pressed against the palate, saliva was passively accumulated in the mouth and was then expectorated into pre-sterilized centrifuge tubes within five minutes. The volunteers were permitted to drink water and rest for one minute before a further five-minute saliva collection was conducted. The collected saliva was placed into clean, sterile centrifuge tubes and was immediately centrifuged at low temperature (4 °C, 4000 rpm, 30 min) to remove suspended particulate matter. The supernatant was then collected, aliquoted into 10 mL portions, and was stored immediately at −80 °C. Prior to each rheological or other assay, the appropriate number of saliva containers were retrieved from storage, warmed to 25 °C in a water bath, and then used.

By mixing the saliva of multiple individuals, the random fluctuations between individuals can be averaged to a certain extent, making the mixed samples more representative.

### 2.8. Simulation of Oral Processing

In a constant-temperature shaking incubator (MJ, Yiheng Scientific Instrument Co., Ltd., Shanghai, China) at 37 °C and 200 rpm, three distinct processing methods were employed to simulate oral processing for three O/W emulsions, thereby characterizing the pivotal role of saliva in oral processing. These methods consisted of: (1) the O/W emulsion was shaken directly for 3 min; (2) the O/W emulsion was mixed with water at a 3:1 ratio and then shaken for 3 min; and (3) the O/W emulsion was mixed with saliva at a 3:1 ratio and then shaken for 3 min.

### 2.9. ζ Potential Analysis

The zeta potential of the three O/W emulsions prepared above and of various mixed systems was analyzed using a laser particle size analyzer (Malvern Instruments Ltd., Malvern, UK, Nano-ZS90). Samples were diluted 400-fold with PBS buffer prior to analysis. Each sample was analyzed in triplicate, and the results were expressed as the mean value.

### 2.10. Analysis of Oil Separation Occurrence

The lipids in the sample were stained using the CLSM method. Following friction, ethanol was employed to remove adhered lipids from the steel ball surface. The solvent was evaporated in a vacuum pump, after which the lipids were resuspended in chloroform. The solution was placed in a quartz cuvette with a path length of 1 mm. At room temperature, emission spectra were acquired using a fluorescence spectrophotometer (FS5, Edinburgh Company, Edinburgh, Scotland, UK) with an excitation wavelength of 530 nm. Spectra were repeatedly collected for three separate samples to determine the adsorption of lipids on the steel ball surface after friction for samples with different emulsifier types and oil concentrations. This characterized lipid release during oral processing.

### 2.11. Statistical Analysis

Each experiment was conducted with a minimum of triplicate replicates to assess data reliability. The mean values and standard deviations of the obtained results were computed using Microsoft Excel and subsequently presented in graphical form. Analysis of significant differences (*p* < 0.05) among samples was conducted using one-way ANOVA, followed by Duncan’s multiple range test.

## 3. Results and Discussion

### 3.1. Effect of Oil Concentration on the Viscosity Characteristics of Casein Systems

**Figure 1:**
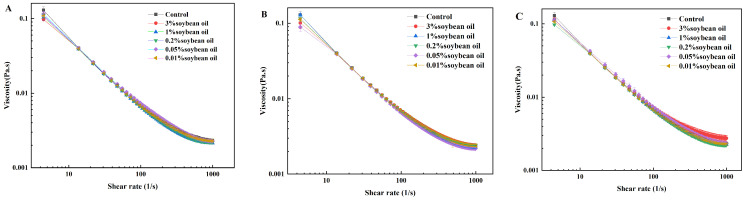
Viscosity changes in three casein emulsions at different oil contents: (**A**) Tween 20 O/W casein emulsion, (**B**) WPI O/W casein emulsion, (**C**) sucrose ester O/W casein emulsion.

Figure 1 compares the viscosity changes in three types of casein emulsions at different oil contents. At shear rates ranging from 0 to 1000 s^−1^, all samples exhibited shear thinning behavior, with emulsion viscosity decreasing significantly at low shear rates. The results indicate that, when compared to casein emulsions prepared with three different emulsifiers, the viscosity of the casein system did not undergo significant changes upon oil addition. Typically, viscosity differences between samples are compared by observing characteristics at a sliding rate of 50 s^−1^. Casein’s rheological and hydrophobic properties facilitate rapid adsorption at the oil–water interface during emulsification, stabilizing oil droplets to prevent flocculation and coalescence [30,31]. Consequently, oil concentration and emulsifier type exert minimal influence on system viscosity. As a natural protein emulsifier, casein has good interfacial activity. It can form a stable adsorption layer at the oil–water interface, which can effectively prevent the coalescence of oil droplets through steric hindrance and electrostatic repulsion, thus maintaining the stability of the emulsion [28]. This is why the oil concentration and emulsifier type have little effect on the viscosity of the system. For Tween 20 O/W casein emulsion, when the oil concentration exceeds 0.2%, the oil droplets increase, but the interface is unstable. High oil accelerates the release of oil, and the film has poor durability; so, it has no significant effect on the friction coefficient [29]. The WPI interface is easily destroyed by salivary enzymes, and the oil concentration has little effect on the viscosity. The low viscosity cannot form an effective film thickness, and the friction coefficient is low under high shear [30].

### 3.2. Effect of Oil Concentration on the Microstructure of Casein Systems

**Figure 2:**
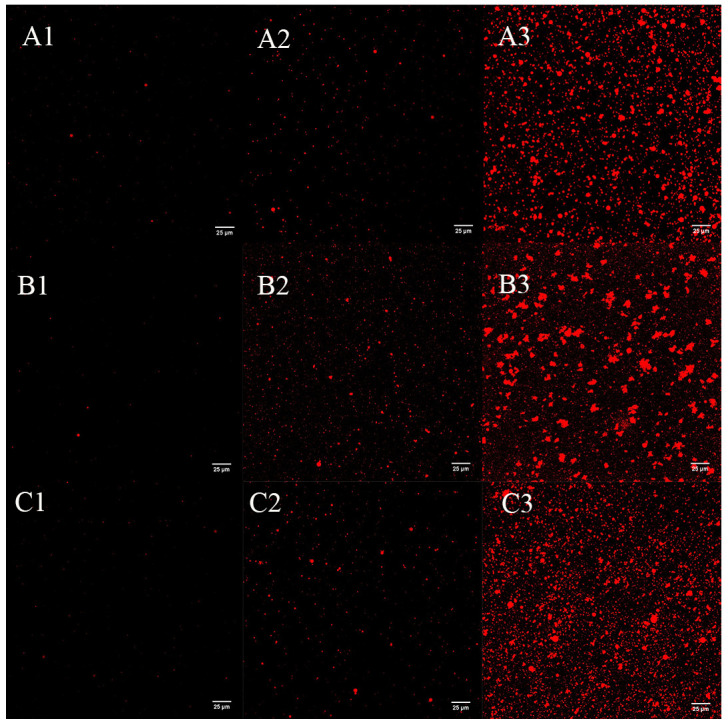
Confocal of three casein emulsions with different oil contents: (**A**) is Tween 20 O/W casein emulsions with different oil contents; ((**A1**) is 0.01% oil, (**A2**) is 0.2% oil, (**A3**) is 3% oil); (**B**) is WPI O/W casein emulsion with different oil content; ((**B1**) is 0.01% oil, (**B2**) is 0.2% oil, (**B3**) is 3% oil); (**C**) is sucrose ester O/W casein emulsion with different oil content; ((**C1**) is 0.01% oil, (**C2**) is 0.2% oil, (**C3**) is 3% oil). Changes with different oil contents shown in Table 1.

Confocal laser scanning microscopy (CLSM) observations were conducted on casein emulsions with varying lipid concentrations. Figure 2 illustrates the distribution patterns of lipids at different concentrations within casein emulsions under various emulsion types. The structural and chemical properties of the hydrophilic/hydrophobic segments in different emulsifiers also influence their interactions with proteins, thereby affecting emulsion stability [32]. As oil content increases, oil droplets exhibit varying degrees of aggregation within the casein emulsion while remaining uniformly dispersed. At a concentration of 0.2%, soybean oil shows slight aggregation, which becomes markedly more pronounced when the concentration rises to 3%. Casein micelles possess excellent emulsifying capacity and surface activity, effectively encapsulating oil that migrates out of the emulsion [33]. From the results of Figure 2(A1,A2), it can be seen that the droplet size increases from 1.684 μm to 3.373 μm, and the particle size of Figure 2(A3) increases to 6.327 μm. Obvious aggregation can be seen.

**Table 1:** Changes in oil droplets of three casein emulsions with different oil contents.

Sample	Droplet Size/μm
Tween 20 + 0.01% oil	1.68 ± 0.01 ^c^
Tween 20 + 0.2% oil	3.37 ± 0.02 ^b^
Tween 20 + 3% oil	6.33 ± 0.02 ^a^
WPI + 0.01% oil	1.70 ± 0.01 ^c^
WPI + 0.2% oil	4.19 ± 0.03 ^b^
WPI + 3% oil	7.33 ± 0.02 ^a^
sucrose ester + 0.01% oil	2.02 ± 0.02 ^c^
sucrose ester 0.2% oil	3.58 ± 0.03 ^b^
sucrose ester + 3% oil	4.53 ± 0.02 ^a^

Different lowercase letters (a–c) indicate significant differences in oil content (*p* < 0.05).

By comparing Figure 2(A3,B3,C3), it can be observed that the casein emulsions prepared using the low-molecular-weight emulsifier Tween 20 and sucrose esters exhibit superior stability with minimal flocculation [34]. The average droplet size in A3, B3, and C3 is 6.327 μm, 7.326 μm and 4.526 μm, respectively. As a protein-based emulsifier, WPI adheres to the surface of oil droplets. Due to the presence of a large quantity of proteins in the casein solution, these proteins interact with WPI, thereby inducing the aggregation of emulsion droplets [35].

### 3.3. Effect of Oil Concentration on the Tribological Properties of Casein Systems

**Figure 3:**
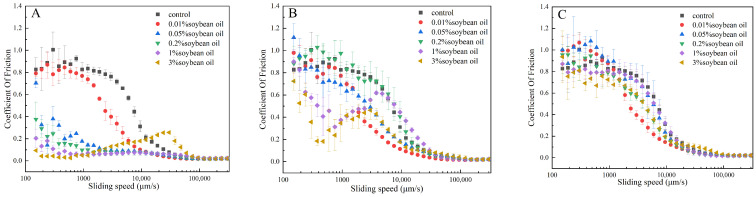
Changes in friction coefficients of three casein emulsions under different oil contents: (**A**) Tween 20 O/W casein emulsion, (**B**) WPI O/W casein emulsion, (**C**) sucrose ester O/W casein emulsion.

The control group refers to the system without oil, and its friction behavior represents the inherent lubrication performance of the emulsion. In the absence of oil, the lubrication performance of the aqueous medium is limited; it is likely to exhibit a high friction coefficient at all speeds, mainly in the boundary lubrication state, because an effective lubricating film cannot be formed to separate the friction surface [31]. Figure 3 illustrates variations in the tribological properties of casein emulsions under differing oil content and emulsifier conditions. The friction coefficient of the control group is higher, and it is in the boundary lubrication state in the whole speed range. The boundary lubrication area of the Tween 20 casein emulsion was reduced when the oil content was 0.2%. The results reveal that in Tween 20 casein emulsions, a critical oil content of 0.2% is observed; beyond this concentration, the coefficient of friction remains largely independent of oil concentration. For this sample, lubrication arises from oil film formation. Most likely, at a 0.2% oil content, the surface becomes saturated with oil droplets, and additional oil droplets no longer contribute to lubrication. At this time, the lubrication mechanism may have changed from boundary lubrication (oil droplets are not enough to completely cover the surface) to mixed lubrication, or even partial hydrodynamic lubrication [31]. In contrast, the WPI casein emulsion exhibits concentration dependence at the highest measured concentration, suggesting that the rolling bearing mechanism is indeed more pertinent to explaining the friction behaviors [36]. WPI O/W casein emulsion at low oil content, due to the emulsifying ability of the protein, even if the oil content is very low, it may form a more stable emulsion, thus entering mixed lubrication and hydrodynamic lubrication earlier. The overall friction coefficient is low. Due to the emulsifying properties of sucrose esters, higher oil content may be required to achieve a similar lubrication effect as Tween 20, and the transition speed is higher. Sucrose esters possess an HLB value of 10, while casein is a natural macromolecular emulsifier. Sucrose esters on the oil surface are readily replaced and encapsulated by substantial amounts of casein, resulting in a friction coefficient that differs little from the control group. Following casein encapsulation, the emulsion droplets exhibit enhanced stability and are less susceptible to disruption. Casein exhibits limited displacement capacity for Tween 20, yet emulsions prepared with it deliver satisfactory lubrication even at lower oil concentrations. For active filler WPI emulsions, casein interacts with the filler, thereby restricting lubricating film formation. Conversely, casein strongly displaces sucrose esters, resulting in enhanced stability but impeding lubricating film formation, thus yielding poorer lubrication performance. As WPI is a protein, the formation of emulsion droplets may be more solid, making them difficult to deform or break, so that it can better play the role of rolling bearings. This means that in the WPI system, the lubrication state may be more dependent on the physical existence of the droplet and its rolling ability, rather than simple oil film spreading. As oil content or speed increases, a partial lubricating film begins to form and bear some of the load, causing the friction coefficient to decrease.

After the WPI casein emulsion reaches a certain concentration, the droplets may also play an auxiliary lubrication role. When the oil content and speed are high enough, the lubricating film completely separates the friction surface, and the friction coefficient is the lowest [32]. At this time, lubrication is mainly determined by the viscous properties of the fluid (oil film or droplet dispersion) [32]. The Tween 20 casein emulsion may have reached the hydrodynamic lubrication state above a 0.2% oil content, while the WPI casein emulsion may reach this state through its unique ‘rolling bearing’ mechanism.

### 3.4. The Effect of Saliva on Zeta Potential Analysis of Emulsion and the Change in Particle Size Distributions

**Figure 4:**
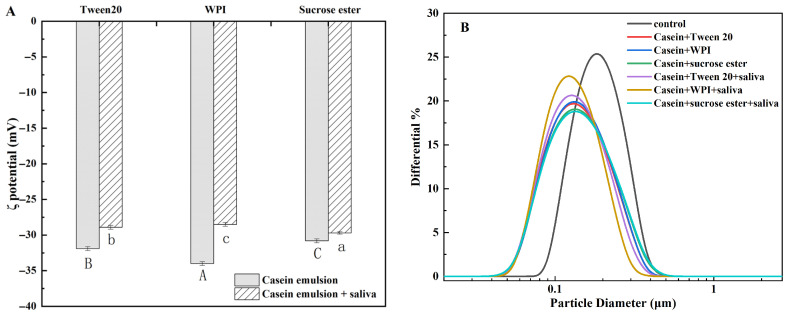
(**A**) Effect of saliva on the ζ-potential of three casein emulsions. (**B**) Effect of saliva on particle size distribution. Different capital letters indicate significant differences (*p* < 0.05) in the potential of three casein emulsions without added saliva. Different lowercase letters denote significant differences (*p* < 0.05) in the potential of the three casein emulsions after adding saliva.

The ζ-potential characterizes the quantity of surface charge carriers on particles within an emulsion [33]. ζ-potential measurements can indicate the potential stability of emulsion systems. After mixing with saliva, the surface charge of the emulsion droplets increased, the absolute value decreased, and the stability decreased. Mucins, lactoferrin, lysozyme, and other salivary proteins can adsorb onto the surfaces of fat droplets within the emulsion thereby imparting charge to these surfaces [34]. A high absolute zeta potential value may induce strong electrostatic repulsion between droplets, preventing aggregation and enhancing emulsion stability [35]. Figure 4A compares zeta potential changes in three casein emulsions after mixing with saliva. The three casein emulsions initially showed negative potentials of −31.9 mV, −34 mV, and −30.8 mV, respectively. Mixing with saliva reduced the absolute potential value, indicating that the state tended to be unstable, and the potential values were −28.9 mV, −28.5 mV, and −29.7 mV, respectively. Due to its naturally self-assembled micellar structure, casein can attract salivary proteins through non-covalent interactions [36]. Combined with the inherent negative potential of saliva, the surface charge of casein emulsions significantly influences their oral processing behavior upon mixing with saliva. Oral processing disrupts the casein emulsion structure, thereby rendering the mixture unstable. The above results indicate that mixing the protein (casein emulsion) system with saliva reduces the stability of the combined system.

The presence of anionic phospho-residues on casein can lead to highly specific binding with Ca^2+^ in saliva, thereby effectively reducing electrostatic repulsion between droplets [37,38]. The decrease in charge density may also reflect changes in the conformation of adsorbed proteins, which in turn alters the stability of the system [39,40].

It can be seen from the particle size distribution map that the particle size of the control group is mainly distributed between 0.1 and 0.3 μm. Most of the other particle sizes are distributed between 0.04 and 0.47 μm. The distribution of the sucrose ester O/W casein emulsion becomes wider after adding saliva, and the distribution of the WPI O/W casein emulsion becomes narrower after adding saliva. When saliva is added, its complex components (such as proteins, mucins, ions, etc.) may interact with the components in the sucrose esters or casein emulsions, causing the particle size distribution to expand in the direction of large size, resulting in a wider distribution [31]. For WPI-stabilized emulsions, surface-active ingredients in saliva (such as certain saliva proteins and peptides) may be further adsorbed to the oil–water interface. These saliva components can reduce the interfacial tension, resulting in a narrower particle size distribution [41]. Saliva is a highly complex biological fluid, which consists of water, electrolytes, mucins, proteins, enzymes (such as salivary amylase, lipase), and microorganisms. The proportion and activity of these components vary from individual to individual and are affected by many factors. The saliva samples used in the experiment were mixed saliva samples from different donors to reduce the difference.

### 3.5. The Effect of Saliva on the Viscosity of Emulsions

**Figure 5:**
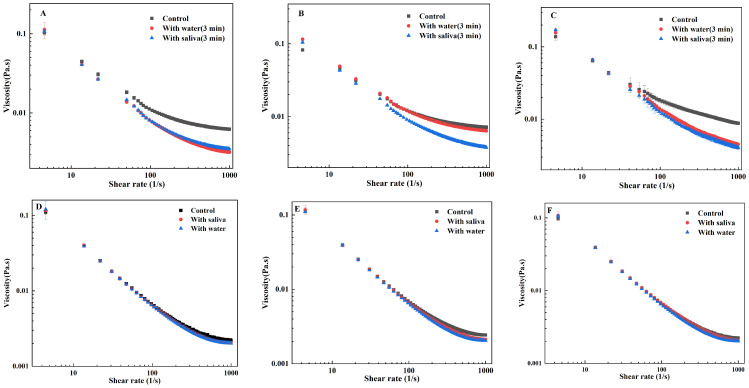
Effect of oral processing on the viscosity of three emulsions ((**A**) Tween 20 O/W emulsion, (**B**) WPI O/W emulsion, (**C**) sucrose ester O/W emulsion). Effect of oral processing method on the viscosity of three casein emulsions: (**D**) Tween 20 O/W emulsion, (**E**) WPI O/W emulsion, (**F**) sucrose ester O/W emulsion.

Certain macromolecular components in saliva can adsorb at the oil–water interface, promoting oil–water compatibility and inducing electrostatic aggregation [42]. Aggregates within the emulsion, when mixed with saliva, contribute to enhancing the viscosity of the food bolus and establishing a distinctive textural perception. Figure 5 illustrates the effect of oral processing on the viscosity of three emulsions. Comparing Figure 5A–C, it is evident that the emulsion prepared with the low-molecular-weight emulsifier Tween 20 exhibits no significant change in viscosity upon mixing with saliva. This may be due to the low-molecular-weight emulsifier-stabilized emulsion (such as Tween 20), the main role of saliva is dilution. Conversely, the viscosities of emulsions prepared with WPI and sucrose esters increase after mixing with saliva, indicating that saliva plays a crucial role in enhancing the viscosity of the emulsion droplet [43]. Polysaccharide colloidal molecules increase the viscosity of the continuous phase, promoting oil droplet stabilization and thereby inhibiting gravity-induced phase separation [44]. Figure 5D–F illustrate the effect of oral processing on the viscosity of the three casein types. When mixed with saliva, the samples exhibited no significant change in viscosity compared to mixtures with water or the samples alone. As this system inherently constitutes an emulsion, dilution effects may be negligible. Furthermore, casein exhibits potent encapsulation capabilities, and its micelles adsorb proteins within saliva, thereby minimizing disruption to the emulsion structure. When proteins in saliva interact with casein micelles, casein micelles may adsorb these salivary proteins through their surface properties (such as electrostatic charge and hydrophobic regions) to form a stable composite structure [45,46]. Consequently, viscosity changes remain insignificant when mixed with either saliva or water. In summary, saliva predominantly exerts a diluting effect on emulsions prepared with the low-molecular-weight surfactant Tween 20. Compared to water, this dilution causes minimal alteration to emulsion viscosity. Although saliva dilutes WPI and sucrose ester emulsions while simultaneously increasing the viscosity of the emulsion mixture, it has no significant effect on the viscosity of casein emulsions.

### 3.6. Analysis of Saliva’s Effect on Emulsion Oil Separation

**Figure 6:**
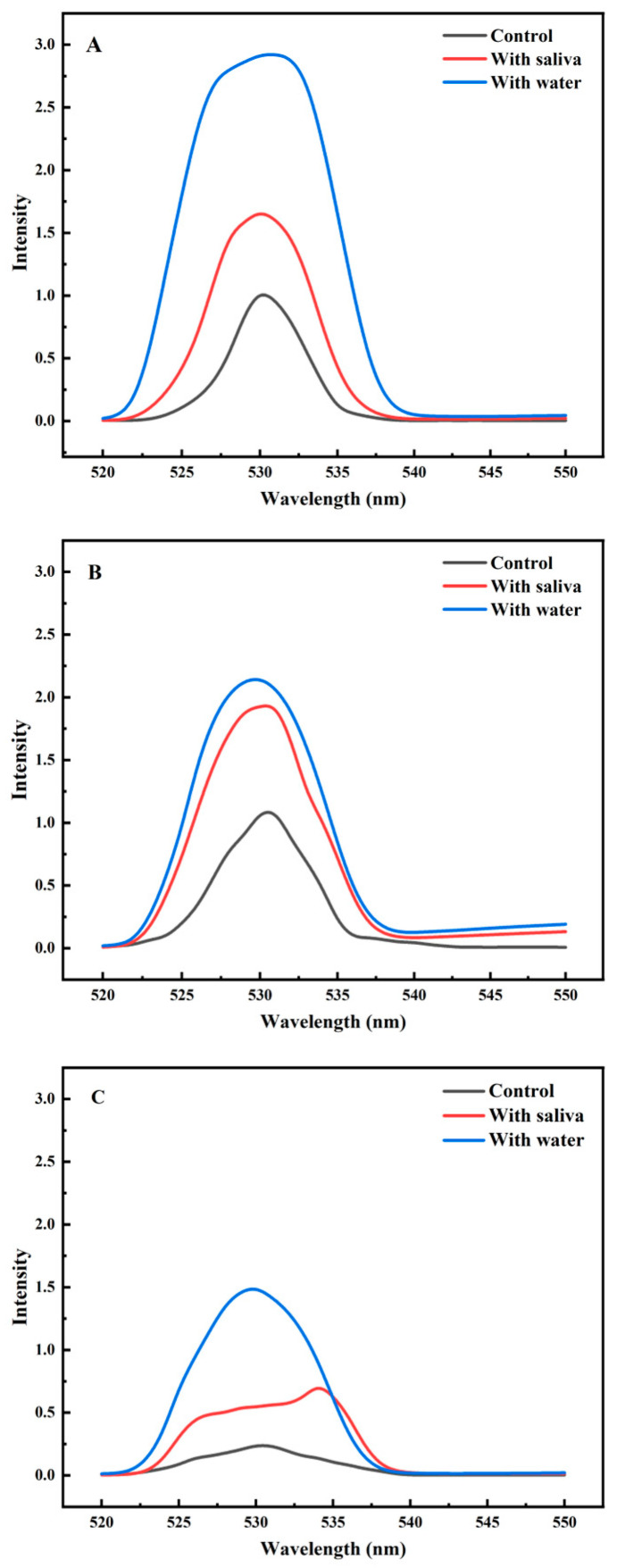
Effect of oral processing on oil release from three casein emulsions: (**A**) Tween 20 O/W casein emulsion, (**B**) WPI O/W casein emulsion, (**C**) sucrose ester O/W casein emulsion.

Figure 6 illustrates the effect of oral processing methods on fat release from three casein emulsions. The results demonstrate that following mixing with saliva and water, the fluorescence intensity on the steel ball surface markedly increased across all three systems. This indicates a greater quantity of fat adhering to the steel ball surface, suggesting a change in the distribution state of fat within the emulsion gel during the sliding friction process. Consistent with the two aforementioned emulsion gels, the role of saliva throughout the oral processing was examined by comparing blank samples with those mixed with water and saliva. In tribology, the plane configuration of steel ball is a common model for studying friction and lubrication. Although it helps to separate specific friction phenomena, it fundamentally simplifies the complex, dynamic and deformable oral environment [47]. The fluorescence intensity of the Tween 20 O/W casein emulsion decreased significantly after saliva treatment, indicating that the oil release was the fastest and most thorough. The strength of WPI O/W casein emulsion decreased significantly after saliva treatment, but it was lower than that of Tween 20, indicating that the release was slow but still significant. The strength of the sucrose ester O/W casein emulsion decreased the least after saliva treatment, indicating that the oil release was the slowest and the least affected by saliva (Figure 6). Figure 6 reveals that saliva significantly reduces fat release compared to water treatment. Consequently, saliva likely functions not only as a diluent but also as an emulsifying agent encapsulating fats within the system [27]. It is hypothesized that salivary proteins, such as mucin, may adsorb at the oil–water interface, forming a stable interfacial film that reduces fat release during friction. Water exhibits stronger dilution effects but lacks emulsifying capacity, making fats more prone to detaching from the emulsion structure and adhering to the steel ball surface. Furthermore, casein emulsions prepared with low-molecular-weight emulsifiers demonstrate superior fat encapsulation, resulting in lower fat release compared to WPI emulsions.

### 3.7. Effect of Saliva on the Microstructure of Emulsions

**Figure 7:**
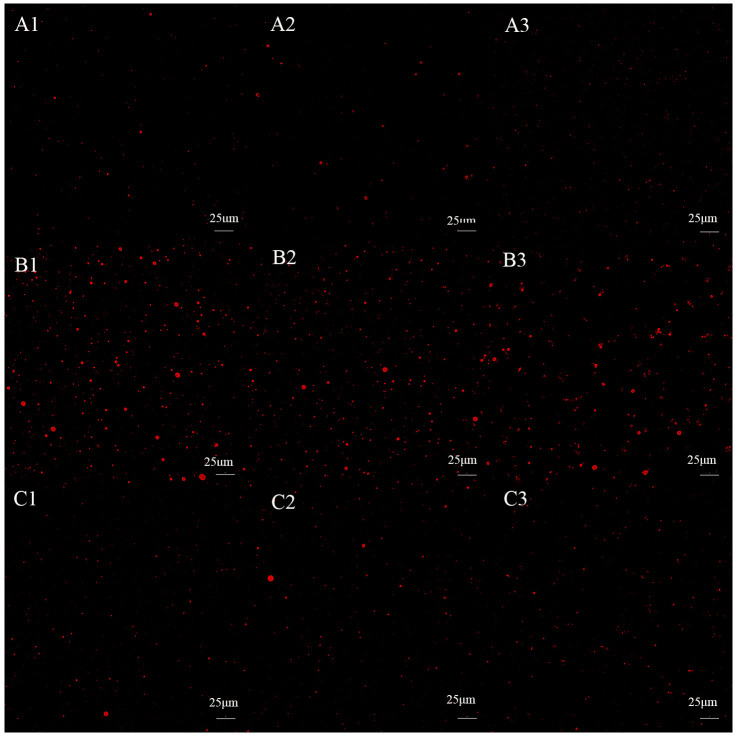
Effects of oral processing on three casein emulsions: (**A**) is Tween 20 O/W casein emulsion ((**A1**) is control, (**A2**) is sample mixed with water, and (**A3**) is sample mixed with saliva); (**B**) is WPI O/W casein emulsion, ((**B1**) is control, (**B2**) is sample mixed with water, and (**B3**) is sample mixed with saliva); (**C**) is sucrose ester O/W casein emulsion, ((**C1**) is control, (**C2**) is sample mixed with water, and (**C3**) is sample mixed with saliva). scale: 25 μm.

The microstructure of three O/W emulsions following oral processing was observed using confocal laser scanning microscopy (CLSM). As shown in Figure 7, within the casein emulsion system prepared using a low-molecular-weight emulsifier, saliva exhibits emulsifying properties relative to water. After mixing with saliva, the size of oil droplets in the casein emulsion decreased significantly. As shown in the results in Table 2, the particle size of Tween 20 decreased from 5.55 ± 0.01 μm to 3.16 ± 0.01 μm after the addition of saliva. The particle size of sucrose esters decreased from 6.92 ± 0.02 to 5.43 ± 0.01 after adding saliva. This indicates that, beyond its diluting effect during oral processing, saliva also emulsifies free lipids, resulting in a more uniform oil droplet distribution. Conversely, in emulsions prepared with macromolecular WPI, the addition of saliva induces aggregation. This phenomenon primarily arises from interactions between salivary proteins and both WPI and casein [36].

### 3.8. Effect of Saliva on the Tribological Properties of Emulsions

Research has confirmed that at the structural level, saliva aids in the breakdown of food structure, facilitates the formation of cohesive boluses, and alters friction within the oral cavity, modifying the sensation of oral oils. That is to say, saliva can act as an emulsifier during oral processing, gradually emulsifying fats [48]. Salivary proteins adsorb to exposed hydrophobic regions at fat interfaces, forming monolayers that thereby create emulsions [48]. At the molecular level, saliva interacts with food components, leading to the formation of new compounds, complexes, and microstructures [49].

**Figure 8:**
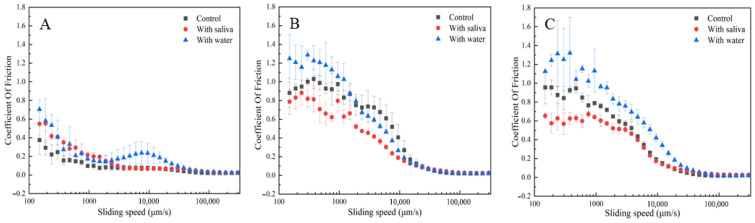
Effect of oral processing on the coefficient of friction of three casein emulsions: (**A**) Tween 20 O/W emulsion, (**B**) WPI O/W emulsion, (**C**) sucrose ester O/W emulsion.

As illustrated in Figure 8, the presence of saliva reduces the friction coefficient of casein emulsions. This reduction primarily stems from saliva’s inherent lubricating properties, which lower the overall friction coefficient of the mixture through interactions with casein. Similar to KGM emulsion gels, Tween 20-casein emulsions inherently possess a low friction coefficient, and the addition of saliva does not significantly alter their lubricating effect.

## 4. Conclusions

This study systematically elucidates the effects of oil concentration and saliva on the oral lubrication behavior of casein emulsion systems. Findings indicate that within the 0.01–3% oil concentration range, the macroscopic viscosity of the system remains largely unchanged. However, microscopic analysis reveals that oil droplet aggregation intensifies with increasing oil concentration. Furthermore, lubrication performance is largely dependent on the type of emulsifier. Ideally, due to the formation of an oil film on the surface, the lubricating effect of Tween 20 emulsion tends to saturate when the oil concentration reaches 0.2%. Conversely, WPI emulsions exhibited continuously enhanced lubrication with increasing oil concentration, consistent with the ‘ball-bearing’ mechanism. Sucrose ester emulsions demonstrated the poorest lubrication due to forming excessively stable interfacial structures with casein. The study indicates that saliva significantly enhances the overall lubrication of the system during oral processing. Moreover, through emulsification, it re-encapsulates fats released during processing, forming finer, more stable oil droplets and thereby reducing fat separation. In summary, emulsifier type serves as the pivotal regulator governing the tripartite synergy between oil, protein, and saliva. Balancing performance metrics, employing WPI as the emulsifier at moderate oil concentrations optimizes sensory texture while achieving health objectives through low fat migration. This approach fully harnesses saliva’s lubricating and emulsifying functions. By balancing performance indicators, WPI is used as an emulsifier at a moderate oil concentration to optimize lubricity, while achieving health goals through low fat migration. Future research should combine quantitative friction data with sensory panel evaluation to establish a direct correlation. Using advanced technology, the formation of interfacial films and droplet aggregation dynamics can be visualized and quantified in real time under simulated oral conditions.

## Figures and Tables

**Table 2:** Effects of oral administration on particle size of three casein emulsions.

Sample	Particle Size/μm
Tween 20	5.55 ± 0.01 ^a^
Tween 20 + water	5.75 ± 0.02 ^a^
Tween 20 + saliva	3.16 ± 0.01 ^b^
WPI	7.81 ± 0.02 ^b^
WPI + water	7.36 ± 0.01 ^b^
WPI + saliva	11.18 ± 0.03 ^a^
sucrose ester	6.92 ± 0.02 ^b^
sucrose ester + water	7.08 ± 0.02 ^a^
sucrose ester + saliva	5.43 ± 0.01 ^b^

Different lowercase letters (a,b) indicate in particle size among different treatments of the same sample (*p* < 0.05).

## Data Availability

The original contributions presented in the study are included in the article. Further inquiries can be directed to the corresponding author.
